# Inflammatory and Epigenetic Biomarkers in Prostate Disease: Current Evidence, Clinical Limitations, and Future Multimarker Strategies

**DOI:** 10.3390/diagnostics16121865

**Published:** 2026-06-16

**Authors:** Imola Donath-Miklos, Romana Olivia Popețiu, Adrian Silviu Crișan, Paula Alexandra Vulciu, Oana Știrbu, Roxana Andra Coman, Cecilia Avram, Denisa Goldiș, Darius Radu Roman, Alexandru Chioreanu, Radmila Anca Bugari, Dana Zdremțan, Simona Maria Borta

**Affiliations:** 1 Department of Biology and Life Science, Faculty of Medicine, “Vasile Goldiș” Western University from Arad, B-dul Revoluției nr. 94-96, 310025 Arad, Romania; avramcici02@yahoo.com (C.A.); timisdenisa333@yahoo.com (D.G.); 2 Department of Internal Medicine, Faculty of Medicine, “Vasile Goldiș” Western University from Arad, B-dul Revoluției nr. 94-96, 310025 Arad, Romania; popetiur@gmail.com (R.O.P.); oanastirbu66@yahoo.com (O.Ș.); simoborta@yahoo.com (S.M.B.); 3 Department of Intensive Care and Emergency Medicine, Faculty of Medicine, “Vasile Goldiș” Western University from Arad, B-dul Revoluției nr. 94-96, 310025 Arad, Romania; adriancrisan74@yahoo.com; 4 Department of Biochemistry, Faculty of Medicine, “Vasile Goldiș” Western University from Arad, B-dul Revoluției nr. 94-96, 310025 Arad, Romania; vulciu.paula@uvvg.ro (P.A.V.); zdremtan.dana@uvvg.ro (D.Z.); 5 Department of Urology, MedLife Humanitas Hospital, Frunzișului Street 77, 400664 Cluj-Napoca, Romania; dr.roxanacoman@yahoo.com; 6 Faculty of Nursing and Health Sciences, Fundamental Discipline, Anatomy, “Iuliu Hațieganu” University of Medicine and Pharmacy, Victor Babeș Street 8, 400347 Cluj-Napoca, Romania; 7 Clinical Laboratory of Medical Analyses, Arad County Emergency Clinical Hospital, Str. Andrényi Károly nr. 2-4, 310037 Arad, Romania; 8 Doctoral School of Biomedical Sciences, Faculty of Medicine and Pharmacy, University of Oradea, Str. Universității nr. 1, 410087 Oradea, Romania; roman.dariusradu@student.uoradea.ro; 9 Department of Otorinolaryngology, Faculty of Medicine, “Vasile Goldiș” Western University from Arad, B-dul Revoluției nr. 94-96, 310025 Arad, Romania; chioreanu.alexandru@uvvg.ro (A.C.); radmilabugariturcin@gmail.com (R.A.B.)

**Keywords:** prostate cancer, benign prostatic hyperplasia, chronic inflammation, YKL-40, mannose-binding lectin, DNA methylation, DNA hydroxymethylation, epigenetic biomarkers, PSA, multiparametric MRI

## Abstract

Prostate disease diagnostics increasingly integrate PSA-derived parameters, molecular assays, risk calculators, and multiparametric MRI, yet important limitations remain in distinguishing benign inflammatory changes from clinically significant prostate cancer and in capturing biological heterogeneity. This narrative review summarizes current evidence on inflammatory and epigenetic biomarkers in prostate disease, focusing on YKL-40, mannose-binding lectin, and global DNA methylation/hydroxymethylation. The reviewed evidence indicates that chronic inflammation, innate immune variability, tumor microenvironment remodeling, and epigenetic dysregulation contribute to prostate disease progression and may provide biological information not fully reflected by conventional diagnostic tools. YKL-40 may reflect inflammatory stromal remodeling and angiogenic activity, mannose-binding lectin may represent innate immune variability, while DNA methylation and hydroxymethylation may indicate systemic molecular adaptation and long-term inflammatory imprinting. However, these biomarkers remain largely investigational and should currently be considered adjunctive biological layers rather than validated standalone diagnostic tools. Future studies should prioritize analytical standardization, prospective prostate-specific validation, and assessment of incremental clinical utility beyond PSA, molecular assays, and mpMRI within clearly defined contexts of use.

## 1. Introduction

Prostate diseases, including benign prostatic hyperplasia (BPH), chronic prostatitis, and prostate cancer (PCa), represent a major source of morbidity and healthcare burden worldwide. Among these conditions, prostate cancer remains one of the most frequently diagnosed malignancies in men and a leading cause of cancer-related mortality, while benign inflammatory and hyperplastic disorders substantially affect quality of life and healthcare utilization. Despite important advances in imaging and molecular diagnostics, the biological heterogeneity of prostate diseases continues to complicate accurate risk stratification and clinical decision-making [1,2,3].

For decades, prostate-specific antigen (PSA) has remained the cornerstone biomarker for prostate disease evaluation and prostate cancer screening. Although PSA testing contributed to earlier detection of clinically significant disease, its limited specificity remains a major clinical challenge. Elevated PSA levels may occur not only in prostate cancer but also in benign prostatic hyperplasia, prostatitis, urinary retention, and other inflammatory or mechanical conditions affecting the prostate. Consequently, PSA-based diagnostic strategies may lead to unnecessary biopsies, overdiagnosis of indolent tumors, and overtreatment, while still failing to fully capture the biological complexity underlying prostate disease progression [4,5,6,7].

The increasing recognition of chronic inflammation as a contributor to prostate tissue remodeling and carcinogenesis has stimulated interest in biomarkers capable of reflecting inflammatory activity and immune dysregulation beyond conventional PSA-centered approaches. Persistent low-grade inflammation has been implicated in epithelial damage, stromal remodeling, angiogenesis, oxidative stress, and genomic instability within the prostate microenvironment. These inflammatory processes may contribute not only to benign prostatic enlargement and lower urinary tract symptoms but also to the initiation and progression of malignant transformation [8,9,10,11,12].

In parallel with inflammatory signaling, epigenetic dysregulation has emerged as another major mechanism involved in prostate disease biology. DNA methylation and hydroxymethylation regulate gene expression, chromatin organization, and cellular differentiation without altering the DNA sequence itself. Chronic inflammatory exposure, aging, oxidative stress, and environmental factors may induce stable epigenetic alterations that contribute to tumor initiation and progression. Importantly, global epigenetic changes can also be detected in peripheral white blood cells, suggesting that blood-based epigenetic biomarkers may provide accessible indicators of systemic biological remodeling associated with prostate disease [13,14,15,16].

Among candidate inflammatory biomarkers, YKL-40 (chitinase-3-like protein 1, CHI3L1) has attracted attention due to its involvement in extracellular matrix remodeling, macrophage activation, angiogenesis, and chronic inflammatory signaling. Similarly, mannose-binding lectin (MBL), a key component of innate immunity and complement activation, represents a biologically plausible marker of interindividual inflammatory susceptibility and immune variability. While these biomarkers have been more extensively investigated in chronic inflammatory and respiratory diseases, their potential translational relevance in prostate pathology remains increasingly recognized [17,18,19,20].

At the same time, contemporary prostate diagnostics are rapidly evolving through the integration of multiparametric magnetic resonance imaging (mpMRI), risk calculators, and molecular urine- or blood-based assays such as PHI, 4Kscore, PCA3, and SelectMDx. Nevertheless, important limitations persist regarding discrimination between indolent and clinically significant disease, prediction of progression, and identification of biologically aggressive phenotypes. In this context, inflammatory and epigenetic biomarkers may provide complementary biological information that is not fully captured by current imaging- and PSA-based approaches [21,22,23,24,25].

**Figure 1:**
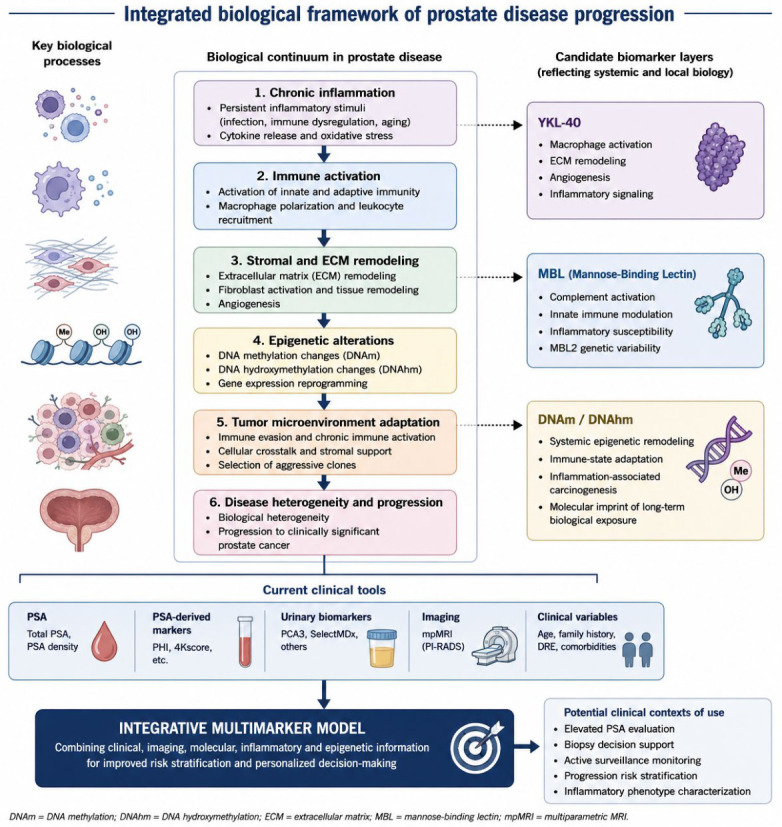
Integrated biological framework of prostate disease progression. Chronic inflammation contributes to stromal remodeling, epigenetic dysregulation, and tumor microenvironment adaptation in prostate disease. YKL-40, MBL, DNAm, and DNAhm may provide complementary biological information beyond conventional diagnostic tools. This figure represents a conceptual framework and not a validated clinical model. Take-home message: Inflammatory and epigenetic biomarkers may complement PSA, molecular assays, and mpMRI by capturing biological processes not reflected by conventional diagnostic tools. Graphical concept development was assisted by ChatGPT (OpenAI, GPT-5 series). All scientific content, figure structure, labeling, interpretation, and final design were created, reviewed, and approved by the authors.

However, despite growing interest in inflammatory and epigenetic biomarkers, most proposed markers remain exploratory and insufficiently validated for routine clinical implementation. Biomarker interpretation is further complicated by biological variability, methodological heterogeneity, and the challenge of establishing clear clinical contexts of use (COU). Therefore, candidate biomarkers should currently be viewed as adjunctive biological layers requiring rigorous analytical and clinical validation rather than as standalone diagnostic tools [26,27,28,29] (Figure 1).

The aim of this narrative review is to summarize current evidence regarding inflammatory and epigenetic biomarkers in prostate-related diseases, with particular emphasis on YKL-40, mannose-binding lectin, and global DNA methylation and hydroxymethylation. Furthermore, this review explores how these biomarkers may contribute to integrative multimarker strategies designed to complement PSA-based diagnostics and improve biological stratification in prostate disease.

## 2. Chronic Inflammation in Prostate Disease Biology

### 2.1. Chronic Inflammation and the Prostate Microenvironment

Chronic inflammation is increasingly recognized as an important biological component of prostate disease development and progression. Histopathological studies frequently demonstrate inflammatory infiltrates in benign prostatic hyperplasia (BPH), prostatitis, and prostate cancer, suggesting that persistent immune activation may contribute to tissue remodeling and disease evolution across the spectrum of prostate pathology [11,30,31,32,33].

Unlike acute inflammatory responses, chronic inflammation is characterized by sustained cytokine signaling, persistent leukocyte recruitment, oxidative stress, and dysregulated tissue repair mechanisms. Within the prostate microenvironment, inflammatory cells, including macrophages, neutrophils, T lymphocytes, and stromal fibroblasts, interact through complex signaling pathways that influence epithelial integrity, extracellular matrix remodeling, angiogenesis, and cellular proliferation [34,35,36].

Several pro-inflammatory mediators, particularly interleukin-6 (IL-6), tumor necrosis factor-α (TNF-α), and nuclear factor κB (NF-κB)-dependent signaling pathways, have been implicated in prostate inflammatory remodeling and carcinogenesis. Persistent activation of these pathways may promote epithelial injury, resistance to apoptosis, stromal reorganization, and genomic instability, thereby creating a biologically permissive environment for neoplastic transformation [37,38,39].

In addition to local tissue effects, chronic inflammatory signaling may contribute to systemic immune dysregulation detectable beyond the prostate itself. Circulating inflammatory mediators and immune-cell alterations reflect the concept that prostate diseases are not exclusively organ-confined biological processes but may also involve broader systemic inflammatory adaptation. This perspective supports growing interest in blood-based inflammatory and molecular biomarkers capable of capturing biologically relevant disease-associated changes [40,41,42].

### 2.2. Inflammation and Age-Related Prostate Remodeling

Age is one of the strongest risk factors for both benign and malignant prostate diseases. The concept of “inflammaging” describes the chronic low-grade inflammatory state associated with aging, characterized by persistent immune activation, altered cytokine profiles, oxidative stress, and progressive tissue remodeling [43,44,45].

In the aging prostate, cumulative inflammatory exposure may contribute to stromal fibrosis, epithelial hyperplasia, vascular alterations, and impaired tissue homeostasis. Age-related immune dysregulation may also influence the prostate tumor microenvironment by promoting chronic cytokine signaling and suppressing effective antitumor immune surveillance [46,47,48].

Importantly, inflammaging provides a biological framework linking chronic inflammation, epigenetic remodeling, and carcinogenesis. Long-term exposure to inflammatory mediators and oxidative stress may induce stable molecular alterations, including changes in DNA methylation and hydroxymethylation patterns, which accumulate over time and may contribute to disease heterogeneity and progression [49,50,51].

### 2.3. Inflammation and Prostate Carcinogenesis

The relationship between chronic inflammation and carcinogenesis has become an important area of investigation in prostate cancer biology. Repeated cycles of tissue injury and repair may facilitate the accumulation of genetic and epigenetic alterations, promoting the transition from benign inflammatory lesions to premalignant and malignant states [52,53,54].

Inflammatory microenvironments can enhance carcinogenesis through multiple mechanisms, including reactive oxygen species generation, DNA damage, cytokine-mediated proliferative signaling, angiogenesis, and immune evasion. Proliferative inflammatory atrophy (PIA) lesions have been proposed as potential intermediates linking chronic inflammation to prostatic intraepithelial neoplasia and prostate cancer development, although the exact sequence of these events remains incompletely understood [55,56,57].

Moreover, inflammation-associated stromal remodeling may influence tumor aggressiveness and metastatic potential by altering extracellular matrix composition and facilitating tumor–stromal interactions. These findings support the concept that inflammatory activity contributes not only to tumor initiation but also to disease progression and biological heterogeneity in prostate cancer [58,59,60].

### 2.4. Implications for Biomarker Development

The multifactorial nature of prostate inflammation highlights the limitations of relying solely on conventional organ-specific biomarkers such as PSA. While PSA reflects prostate epithelial disruption and glandular activity, it provides limited information regarding immune activation, inflammatory remodeling, or systemic biological adaptation [4,5,6,7].

Consequently, increasing attention has focused on biomarkers capable of reflecting complementary dimensions of prostate disease biology, including inflammatory mediators, innate immune regulators, and epigenetic alterations. Integrative biomarker strategies may provide a more biologically informative framework for risk stratification and disease characterization, particularly in clinically heterogeneous settings where PSA alone offers insufficient specificity [26,27,28,29].

## 3. Current Diagnostic Landscape and Unmet Needs in Prostate Disease

### 3.1. Prostate-Specific Antigen and Conventional Diagnostic Approaches

Prostate-specific antigen (PSA) remains the most widely used biomarker in prostate disease screening and diagnostic evaluation. Since its introduction into clinical practice, PSA testing has substantially influenced prostate cancer detection by enabling identification of disease at earlier stages. However, despite its widespread clinical utility, PSA lacks sufficient specificity for distinguishing malignant disease from benign inflammatory and hyperplastic conditions of the prostate [61,62,63].

Elevated serum PSA levels may occur in benign prostatic hyperplasia, prostatitis, urinary retention, catheterization, or other nonmalignant conditions associated with epithelial disruption and inflammatory activity. Consequently, PSA-based screening strategies may contribute to unnecessary prostate biopsies, overdiagnosis of indolent tumors, and overtreatment of clinically insignificant disease [64,65].

To improve diagnostic performance, several PSA-derived parameters have been incorporated into clinical practice. Free PSA percentage (%fPSA) has been used to improve discrimination between benign and malignant disease, particularly in patients with intermediate PSA levels. Lower free PSA fractions are generally associated with increased probability of prostate cancer, although overlap between benign and malignant conditions remains substantial [66,67].

Similarly, PSA density (PSAD), calculated by relating PSA levels to prostate volume, attempts to account for benign gland enlargement and improve risk stratification. While PSA density demonstrates improved predictive value compared with PSA alone in certain clinical contexts, its interpretation remains influenced by imaging variability and overlapping biological characteristics among prostate diseases [68,69,70,71].

### 3.2. Emerging Blood- and Urine-Based Biomarkers

The limitations of conventional PSA-based diagnostics have stimulated the development of additional blood- and urine-based biomarkers aimed at improving detection of clinically significant prostate cancer while reducing unnecessary biopsies.

The Prostate Health Index (PHI), which combines total PSA, free PSA, and [-2]proPSA into a mathematical model, has demonstrated improved specificity for clinically significant prostate cancer compared with PSA alone. Similarly, the 4Kscore integrates four kallikrein markers with clinical variables to estimate the probability of high-grade prostate cancer [72,73,74,75].

Urine-based molecular assays have also emerged as promising adjunctive tools. Prostate cancer antigen 3 (PCA3), a noncoding RNA overexpressed in prostate cancer tissue, has been investigated primarily as a post-digital rectal examination urinary biomarker. SelectMDx combines urinary mRNA expression markers with clinical parameters to improve identification of patients at risk for clinically significant disease [73,74,75,76].

Although these assays improve risk stratification compared with PSA alone, their clinical performance remains influenced by patient selection, disease prevalence, and integration with imaging findings. Furthermore, most currently available biomarkers primarily focus on cancer detection rather than on capturing the broader biological complexity of inflammation-associated prostate remodeling and systemic molecular dysregulation [76,77,78,79].

Recent interest has also focused on emerging blood-based liquid biopsy approaches aimed at improving noninvasive detection and biological characterization of prostate cancer. Among these, circulating tumor cell (CTC)-based platforms such as TruBlood have been investigated as potential adjunctive tools for identifying clinically significant prostate cancer through enrichment and molecular characterization of tumor-associated circulating cells. Preliminary studies have reported promising diagnostic performance; however, these technologies currently remain investigational and require further large-scale prospective validation before integration into routine clinical practice [80,81].

### 3.3. Multiparametric MRI and Risk-Stratified Diagnostic Pathways

The integration of multiparametric magnetic resonance imaging (mpMRI) into prostate diagnostic algorithms has significantly changed contemporary clinical practice. mpMRI improves visualization of suspicious lesions, guides targeted biopsy strategies, and enhances detection of clinically significant prostate cancer while reducing unnecessary biopsy procedures [66,67,68,69,70].

Risk-adapted diagnostic models increasingly combine PSA-related variables, imaging findings, family history, age, and molecular biomarkers into predictive risk calculators designed to optimize biopsy decisions and individualized patient management. Such approaches reflect the transition from single-marker diagnostics toward more integrated and biologically informed clinical decision-making [82,83,84].

Contemporary diagnostic pathways increasingly support MRI-first or risk-adapted approaches integrating serum and urinary biomarkers with imaging findings. Nevertheless, despite substantial advances in imaging and molecular diagnostics, important unmet clinical needs remain. Current diagnostic tools still demonstrate limitations in differentiating indolent from biologically aggressive disease, predicting progression during active surveillance, and distinguishing inflammation-associated PSA elevation from clinically significant malignancy [61,70,75,78,79].

### 3.4. Unmet Biological Needs and the Rationale for Novel Biomarkers

Most currently implemented prostate biomarkers primarily reflect glandular disruption, tumor-associated protein expression, or structural imaging abnormalities. Comparatively less attention has focused on biomarkers capable of reflecting chronic inflammatory remodeling, innate immune variability, systemic immune activation, and long-term molecular reprogramming associated with prostate disease biology [26,27,28,29].

Increasing evidence suggests that chronic inflammation, immune dysregulation, and epigenetic alterations contribute substantially to prostate disease heterogeneity and progression. Consequently, inflammatory and epigenetic biomarkers may provide complementary biological information not fully captured by PSA, molecular urine assays, or imaging-based approaches alone [30,31,49,50].

**Figure 2:**
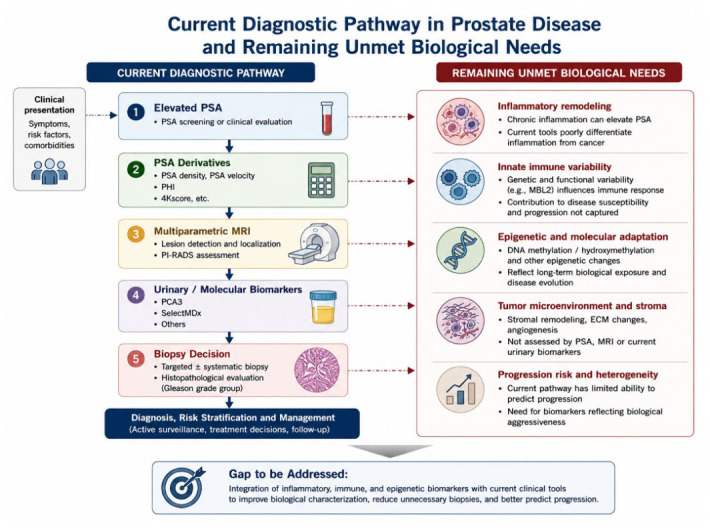
Current diagnostic pathway and remaining unmet biological needs in prostate disease. Contemporary prostate diagnostics integrate PSA, molecular assays, mpMRI, and risk calculators to support biopsy decisions and risk stratification. However, chronic inflammation, immune dysregulation, and long-term molecular remodeling remain incompletely captured by current diagnostic approaches. This figure illustrates the rationale for incorporating inflammatory and epigenetic biomarkers into future integrative diagnostic frameworks. Take-home message: Inflammatory and epigenetic biomarkers may provide complementary biological information beyond conventional diagnostic tools and improve future multimarker risk assessment strategies. Graphical concept development was assisted by ChatGPT (OpenAI, GPT-5 series). All scientific content, figure structure, labeling, interpretation, and final design were created, reviewed, and approved by the authors.

Within this context, biomarkers such as YKL-40, mannose-binding lectin, and global DNA methylation/hydroxymethylation profiles represent biologically plausible candidates for integrative multimarker models aimed at improving biological stratification and refining prostate disease characterization. Nevertheless, these candidate biomarkers remain largely exploratory, and rigorous validation studies are still required before routine clinical implementation can be considered [26,27,28,29] (Figure 2).

## 4. YKL-40 in Prostate Diseases

### 4.1. Biological Role of YKL-40 in Inflammation and Tissue Remodeling

YKL-40, also known as chitinase-3-like protein 1 (CHI3L1), is a secreted glycoprotein produced by several cell types involved in inflammatory and remodeling processes, including activated macrophages, neutrophils, fibroblasts, endothelial cells, and tumor-associated stromal cells. Although YKL-40 lacks enzymatic chitinase activity, it has been implicated in extracellular matrix remodeling, cell migration, angiogenesis, macrophage activation, and chronic inflammatory signaling. These biological properties make YKL-40 relevant to diseases characterized by persistent tissue injury and repair, including cancer-associated inflammatory microenvironments [17,85,86].

In cancer biology, YKL-40 has been associated with tumor–stromal interactions, angiogenic signaling, macrophage polarization, and immune-modulatory processes. Experimental and clinical studies suggest that CHI3L1/YKL-40 expression may reflect interactions between malignant cells and the surrounding microenvironment rather than tumor-cell activity alone. Therefore, in prostate disease, YKL-40 is best interpreted as a candidate marker of inflammation-associated remodeling and tumor microenvironment activity rather than as a prostate-specific diagnostic biomarker [17,86,87,88,89].

### 4.2. YKL-40, Tumor Microenvironment, and Stromal Remodeling in Prostate Cancer

The prostate tumor microenvironment consists of epithelial cells, cancer-associated fibroblasts, immune cells, endothelial cells, extracellular matrix components, and soluble inflammatory mediators. Dynamic interactions between these elements influence tumor initiation, progression, immune evasion, angiogenesis, and therapeutic resistance. In this context, biomarkers reflecting stromal activation and inflammatory remodeling may provide complementary information to markers derived primarily from epithelial prostate cells [90,91,92].

YKL-40 may be biologically relevant in this setting because its known functions overlap with several processes involved in prostate cancer progression, including macrophage activation, extracellular matrix remodeling, endothelial activation, and angiogenesis. Previous studies have reported increased YKL-40 expression in advanced prostate cancer tissues and suggested associations with more aggressive disease features. However, the available evidence remains limited and heterogeneous, and YKL-40 should not currently be considered a validated clinical biomarker for prostate cancer diagnosis or prognosis [93,94,95].

From a translational perspective, YKL-40 may be more useful as part of a broader inflammatory and stromal biomarker framework than as a standalone diagnostic marker. Its potential value may lie in capturing a biological layer related to remodeling, macrophage-associated inflammation, and angiogenic activity, which is not directly reflected by PSA, PSA-derived parameters, or imaging findings. Nevertheless, prostate-specific prospective validation studies are required to determine whether YKL-40 provides incremental diagnostic or prognostic information beyond established tools [26,27,28,29,90,91,92,93,94,95].

### 4.3. Clinical Limitations and Future Directions

The major limitation of YKL-40 as a biomarker is its lack of disease specificity. Circulating YKL-40 concentrations may be influenced by age, systemic inflammation, cardiovascular disease, metabolic disorders, chronic pulmonary disease, and malignancies outside the prostate. This broad biological responsiveness limits its direct diagnostic specificity and requires careful interpretation in patients with multimorbidity [17,85,96].

Consequently, future prostate-focused research should evaluate YKL-40 within clearly defined contexts of use, such as discrimination of inflammatory PSA elevation from clinically significant prostate cancer, characterization of inflammatory or stromal tumor phenotypes, progression risk during active surveillance, or prediction of treatment response. At present, YKL-40 remains a biologically plausible candidate marker requiring standardized assays, prostate-specific cohorts, and demonstration of incremental utility before clinical implementation can be considered [26,27,28,29,96].

## 5. Mannose-Binding Lectin and Innate Immune Variability

### 5.1. Mannose-Binding Lectin and the Lectin Complement Pathway

Mannose-binding lectin (MBL) is a soluble pattern-recognition molecule of the innate immune system that recognizes carbohydrate structures on microbial and altered host surfaces. After ligand binding, MBL activates the lectin pathway of complement through MBL-associated serine proteases, contributing to opsonization, inflammatory signaling, and immune clearance. Beyond antimicrobial defense, complement activation can also modulate tissue inflammation, immune-cell recruitment, and tumor microenvironment dynamics [97,98,99].

In cancer biology, the complement system has a context-dependent role. Complement activation may support immune surveillance and tumor-cell clearance in some settings, but chronic or dysregulated activation can also promote inflammation, angiogenesis, immune suppression, and tumor progression. Therefore, MBL-related complement activity should be interpreted as a regulatory component of innate immune balance rather than as uniformly protective or harmful [100,101,102].

### 5.2. MBL2 Genetic Variability and Inflammatory Susceptibility

Circulating MBL concentrations and functional activity vary substantially between individuals, largely because of polymorphisms in the MBL2 gene. Structural and promoter variants influence MBL synthesis, oligomerization, serum concentration, and complement-activating capacity. As a result, genetically determined MBL variability may contribute to differences in inflammatory susceptibility, infection risk, immune activation, and tissue response to chronic inflammatory stimuli [103,104,105].

In prostate disease, direct evidence regarding MBL or MBL2 as diagnostic or prognostic biomarkers remains limited. However, the biological plausibility is supported by the broader role of complement activation and innate immune regulation in chronic inflammation and cancer microenvironments. MBL2 variability may theoretically influence prostate inflammatory phenotypes, local immune activation, or systemic inflammatory responsiveness, but these hypotheses require prostate-specific validation [100,101,102,103,104,105].

### 5.3. Translational Relevance in Prostate Disease

The relevance of MBL in prostate-related pathology should currently be framed as mechanistic and exploratory. Chronic inflammation contributes to prostate remodeling and carcinogenesis, and complement signaling may participate in immune-cell recruitment, inflammatory amplification, and tumor–stroma interactions. Within this framework, MBL could represent one component of innate immune variability that modifies individual inflammatory responses rather than a direct disease marker [30,92,100,101,102].

Compared with PSA-derived markers or urine-based molecular assays, MBL is unlikely to function as an organ-specific diagnostic marker. Its potential value, if confirmed, would more likely emerge in multimarker models integrating inflammatory mediators, innate immune variability, epigenetic alterations, and conventional clinical variables. Such models could help characterize biological heterogeneity in prostate disease, but clinical utility has not yet been established [26,27,28,29].

### 5.4. Current Limitations

Several limitations restrict the immediate clinical applicability of MBL assessment. These include wide interindividual variability, population-specific genetic distributions, context-dependent biological effects, assay heterogeneity, and limited prostate-specific clinical data. Moreover, both low and high MBL activity may be biologically relevant depending on clinical context, making interpretation more complex than a simple deficiency-risk model [97,98,99,100,101,102,103,104,105].

Therefore, MBL and MBL2 should currently be considered candidate markers of innate immune variability rather than validated biomarkers for prostate disease diagnosis. Future studies should evaluate circulating MBL levels and MBL2 genotypes in well-defined prostate cohorts, ideally in combination with inflammatory, epigenetic, imaging, and clinical parameters (Table 1).

## 6. Epigenetic Biomarkers in Prostate Diseases

### 6.1. DNA Methylation and Hydroxymethylation in Chronic Inflammatory and Neoplastic Processes

Epigenetic mechanisms regulate gene expression without altering the underlying DNA sequence and represent an important interface between environmental exposure, chronic inflammation, aging, and carcinogenesis. Among these mechanisms, DNA methylation (DNAm) and DNA hydroxymethylation (DNAhm) are particularly relevant because they influence chromatin organization, transcriptional activity, genomic stability, and cellular differentiation [106,107,108,109].

DNA methylation typically involves the addition of a methyl group to cytosine residues within CpG dinucleotides and is generally associated with transcriptional repression. DNA hydroxymethylation, generated through ten-eleven translocation (TET) enzymes, represents both an intermediate step in active DNA demethylation and an independent epigenetic regulatory mark [107,108].

Chronic inflammatory signaling may substantially influence epigenetic regulation. Cytokine-mediated activation of NF-κB, JAK/STAT, oxidative stress pathways, and metabolic alterations can modulate DNA methyltransferase and TET enzyme activity, thereby contributing to persistent molecular remodeling. These epigenetic alterations may accumulate over time and participate in inflammation-associated carcinogenesis and tumor progression [110,111,112].

In prostate disease, epigenetic dysregulation has been implicated in epithelial transformation, stromal remodeling, tumor heterogeneity, and progression toward clinically aggressive phenotypes. Both global and gene-specific methylation abnormalities have been described in benign and malignant prostate conditions, supporting the concept that epigenetic remodeling represents a biologically important layer of prostate disease evolution [113,114,115,116].

DNA methylation and DNA hydroxymethylation represent two closely related but functionally distinct epigenetic modifications involved in prostate disease development and progression. In general, promoter hypermethylation is associated with transcriptional repression through chromatin condensation and reduced accessibility of transcription factors to regulatory genomic regions. Consequently, aberrant hypermethylation frequently results in the silencing of tumor suppressor genes and disruption of normal cellular homeostasis. In contrast, DNA hydroxymethylation (5-hydroxymethylcytosine, 5hmC), generated through TET enzyme-mediated oxidation of 5-methylcytosine, is often associated with transcriptionally permissive chromatin states and active gene expression, although its biological effects remain context-dependent and may vary according to genomic location and disease stage [117,118].

Several genes implicated in prostate carcinogenesis exhibit characteristic methylation abnormalities. Among the best-established examples is GSTP1, whose promoter hypermethylation is considered one of the most frequent epigenetic alterations in prostate cancer and has been proposed as a diagnostic biomarker. Additional tumor suppressor genes frequently affected by promoter hypermethylation include APC, involved in Wnt signaling regulation, and RASSF1A, which participates in cell-cycle control, apoptosis, and genomic stability. Methylation-mediated silencing of these genes may contribute to enhanced cellular proliferation, impaired DNA damage responses, and tumor progression. Furthermore, alterations involving CDKN2A (p16) and components of the PTEN-associated signaling network have also been linked to aggressive disease phenotypes and adverse clinical outcomes [117,119,120].

Beyond conventional DNA methylation, emerging evidence suggests that hydroxymethylation may provide additional biological and clinical information. Global loss of 5hmC has been consistently observed in advanced prostate cancer and is thought to reflect progressive disruption of normal epigenetic regulation. Altered TET enzyme activity and locus-specific changes in hydroxymethylation patterns have been associated with dysregulated expression of genes involved in cellular differentiation, immune regulation, androgen signaling, and tumor progression. These findings suggest that hydroxymethylation may not only serve as a biomarker of disease evolution but may also actively participate in prostate cancer pathogenesis through dynamic modulation of gene expression programs [118].

Collectively, current evidence supports the concept that DNA methylation and hydroxymethylation are not merely passive molecular markers but biologically active processes that influence prostate disease initiation, progression, and therapeutic responsiveness. Integration of methylation and hydroxymethylation profiling into future biomarker panels may improve risk stratification and facilitate more precise, biology-informed clinical decision-making [117,118,120].

### 6.2. White Blood Cell Epigenetics as a Systemic Biomarker Layer

Peripheral white blood cells (WBCs) provide an accessible source for evaluating systemic epigenetic alterations associated with chronic inflammatory states and cancer biology. Unlike tissue biopsies, blood-based epigenetic assessment offers minimally invasive sampling and potential longitudinal applicability in disease monitoring and risk stratification [121,122,123].

Importantly, blood-based DNAm and DNAhm profiles should not be interpreted as direct surrogates of prostate tissue epigenetics without careful qualification. Peripheral leukocyte epigenetic signatures integrate systemic inflammatory exposure, immune-cell composition, aging, environmental influences, and chronic immune activation. Therefore, WBC epigenetic alterations may reflect a broader systemic immune-state phenotype rather than organ-specific molecular events alone [122,123,124].

Nevertheless, this systemic character may also represent a potential advantage. Because chronic inflammation and immune dysregulation contribute to prostate disease biology, circulating epigenetic alterations detectable in leukocytes could provide complementary information regarding cumulative inflammatory burden and long-term molecular adaptation not captured by conventional organ-specific biomarkers [110,111,112].

Global methylation and hydroxymethylation measurements in circulating leukocyte DNA therefore represent a biologically plausible candidate biomarker layer that may complement PSA-based diagnostics and imaging findings, particularly in contexts where inflammatory remodeling and systemic immune adaptation contribute to disease heterogeneity [16,106,107,108,109].

### 6.3. Evidence of Epigenetic Alterations in Prostate Disease

Accumulating evidence suggests that epigenetic dysregulation contributes to prostate carcinogenesis and disease progression. Aberrant methylation patterns have been associated with altered expression of tumor suppressor genes, inflammatory signaling pathways, androgen receptor regulation, and tumor microenvironment remodeling [113,114,115,116,125].

Several studies have investigated systemic epigenetic alterations detectable in peripheral blood cells. Exploratory analyses evaluating global DNA methylation and hydroxymethylation in leukocyte DNA demonstrated measurable differences between patients with prostate cancer, benign prostate disease, and healthy controls, suggesting that circulating epigenetic alterations may reflect biologically relevant disease-associated processes [126,127,128].

Blood-based epigenetic biomarkers have attracted increasing interest because they may provide molecular information beyond conventional PSA measurements and structural imaging findings. In particular, circulating methylation signatures may reflect long-term inflammatory exposure, immune adaptation, and systemic biological remodeling associated with prostate disease progression [121,122,123,129].

Importantly, current evidence remains preliminary and heterogeneous. Differences in analytical methods, cohort selection, biological confounders, and outcome definitions complicate direct comparison between studies. Consequently, epigenetic biomarkers should currently be interpreted as investigational molecular tools requiring rigorous validation rather than clinically established diagnostic markers [130,131,132].

### 6.4. Clinical Implications and Translational Perspectives

The potential translational value of epigenetic biomarkers lies in their ability to integrate chronic inflammatory exposure, immune dysregulation, aging-related molecular remodeling, and tumor-associated biological adaptation into measurable systemic signatures [109,110,111,112].

In prostate disease, blood-based epigenetic biomarkers could theoretically contribute to several clinically relevant contexts of use. These include support for biopsy decision-making in patients with elevated PSA, refinement of risk stratification models, characterization of inflammatory phenotypes, and identification of patients at increased risk of progression during active surveillance [78,79,80,81,82,133].

Epigenetic markers may also complement contemporary MRI-based diagnostic pathways by adding a molecular layer reflecting systemic biological remodeling rather than solely structural imaging abnormalities. Such integration could potentially improve discrimination between inflammation-associated PSA elevation and clinically significant malignancy, although this hypothesis requires prospective validation [61,66,67,68,69,70].

At present, however, the clinical utility of DNAm and DNAhm measurements remains limited by methodological heterogeneity, biological variability, lack of standardized thresholds, and insufficient prospective prostate-specific validation. Future studies should prioritize harmonized analytical platforms, longitudinal cohort designs, and assessment of incremental value beyond PSA, mpMRI, and currently available molecular assays [130,131,132,133,134,135] (Table 2).

## 7. Integrative Multimarker Models in Prostate Disease

### 7.1. Rationale for Integrative Biomarker Strategies

The biological complexity of prostate disease cannot be fully captured by a single biomarker. PSA primarily reflects prostate epithelial disruption and glandular activity, while mpMRI focuses on structural imaging abnormalities. Neither approach fully characterizes inflammatory remodeling, systemic immune activation, stromal interactions, or long-term molecular reprogramming associated with disease heterogeneity [61,62,63,64,65,66,67,68,69,70].

**Figure 3:**
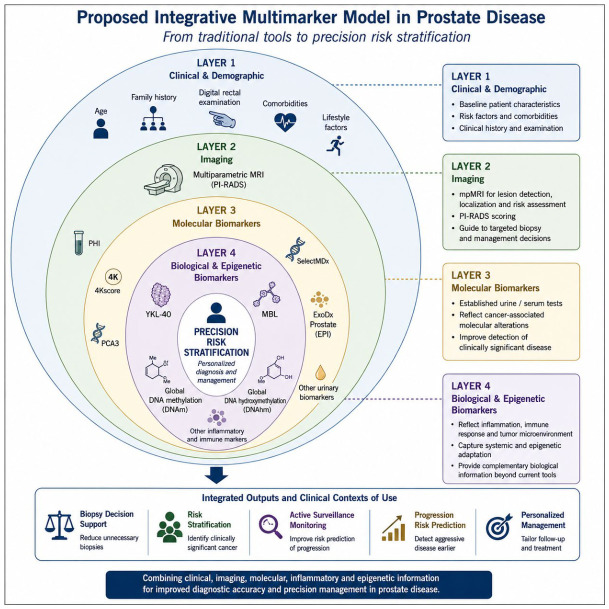
Conceptual integrative multimarker model for biologically informed prostate disease stratification. Contemporary prostate diagnostics integrate clinical variables, PSA-derived parameters, imaging findings, and molecular assays to improve risk assessment. Inflammatory and epigenetic biomarkers may provide additional information on immune activation, chronic inflammatory burden, stromal remodeling, and long-term molecular adaptation. This figure illustrates a conceptual multimarker framework rather than a validated clinical algorithm. Take-home message: Future prostate disease stratification may benefit from integrating clinical, imaging, molecular, inflammatory, and epigenetic biomarker layers into a unified diagnostic framework. Graphical concept development was assisted by ChatGPT (OpenAI, GPT-5 series). All scientific content, figure structure, labeling, interpretation, and final design were created, reviewed, and approved by the authors.

Consequently, increasing attention has focused on multimarker strategies integrating complementary biological layers into a unified diagnostic framework. In prostate disease, this may involve combining PSA-related parameters, imaging findings, inflammatory mediators, innate immune variability, and epigenetic alterations to improve biological characterization and individualized risk assessment [80,81,82] (Figure 3).

### 7.2. Potential Clinical Contexts of Use

One of the most clinically relevant contexts is the evaluation of patients with elevated PSA levels and indeterminate risk profiles. In this setting, inflammatory and epigenetic biomarkers could theoretically provide complementary biological information helping differentiate inflammation-associated PSA elevation from clinically significant prostate cancer [63,64,65,133].

Another important context involves biopsy decision support. Current MRI-guided and biomarker-assisted pathways have reduced unnecessary biopsies; however, uncertainty persists in intermediate-risk cases. Integrative models combining PSA density, mpMRI findings, inflammatory markers, and epigenetic signatures may improve patient stratification and reduce diagnostic ambiguity [66,67,68,69,70,72,73,74,75,76,77,136].

Active surveillance also represents a potential area of application. Monitoring biologically indolent versus progressive disease remains challenging despite advances in imaging and molecular diagnostics. Systemic inflammatory and epigenetic biomarkers may theoretically provide additional information regarding progression-associated biological remodeling during surveillance protocols [78,79,80,81,82,137,138].

**Figure 4:**
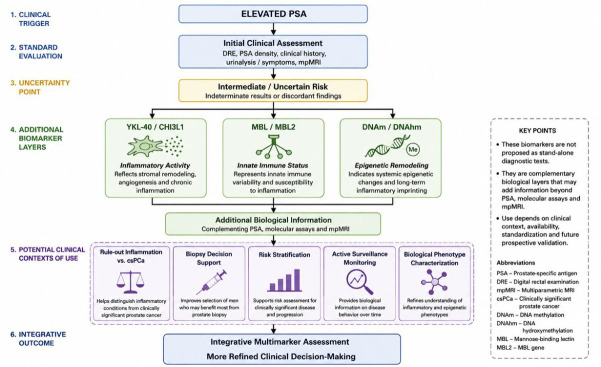
Diagnostic decision map illustrating potential clinical contexts of use for inflammatory and epigenetic biomarkers in prostate disease. Take-home message: Inflammatory and epigenetic biomarkers are not proposed as standalone diagnostic tools but as complementary biological layers that may improve risk stratification and support clinical decision-making when integrated with PSA, molecular assays, and multiparametric MRI. Graphical concept development was assisted by ChatGPT (OpenAI, GPT-5 series). All scientific content, figure structure, labeling, interpretation, and final design were created, reviewed, and approved by the authors.

Finally, integrative biomarker models may help characterize inflammatory or stromal phenotypes within prostate disease. Because chronic inflammation and tumor microenvironment remodeling contribute to disease heterogeneity, multimarker approaches incorporating inflammatory and epigenetic alterations could support more biologically informed disease classification [17,85,86,87,88,89,90,91,92,93,94,95,96,97,98,99,100,101,102,103,104,105] (Figure 4).

### 7.3. Current Limitations and Future Directions

Despite strong biological rationale, integrative multimarker models remain largely investigational. Several limitations currently restrict clinical implementation, including assay variability, cohort heterogeneity, confounding effects of aging and comorbidities, and insufficient prospective validation [130,131,132,133,135,139].

An additional challenge involves determining whether novel biomarkers provide clinically meaningful incremental value beyond existing tools such as PSA density, PHI, 4Kscore, SelectMDx, and mpMRI. Demonstration of biological plausibility alone is insufficient for clinical translation; candidate biomarkers must improve diagnostic performance, decision-making, or patient outcomes within clearly defined contexts of use [80,81,82].

**Figure 5:**
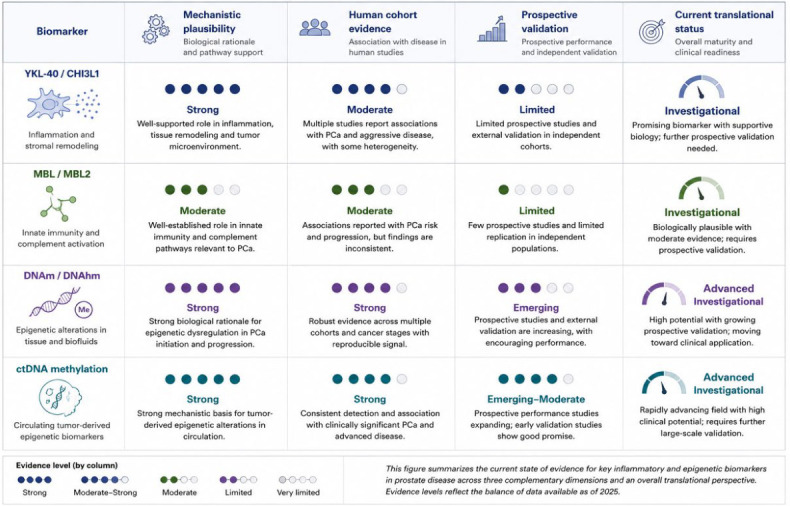
Evidence tiers for inflammatory and epigenetic biomarkers in prostate disease. Take home message: The translational maturity of inflammatory and epigenetic biomarkers varies substantially, with mechanistic evidence generally exceeding prospective clinical validation. Future clinical implementation will require robust prospective studies and integration with established diagnostic pathways. Graphical concept development was assisted by ChatGPT (OpenAI, GPT-5 series). All scientific content, figure structure, labeling, interpretation, and final design were created, reviewed, and approved by the authors.

Future prostate-focused studies should therefore prioritize prospective multimodal validation strategies integrating imaging, inflammatory biomarkers, epigenetic profiling, and clinical variables in large, well-characterized cohorts. Such approaches may help transition prostate diagnostics from predominantly anatomy-based assessment toward more biologically informed precision stratification models [133,134,135,137,138] (Figure 5).

## 8. Challenges and Translational Barriers

### 8.1. Analytical Validity and Assay Standardization

The successful clinical implementation of inflammatory and epigenetic biomarkers in prostate disease depends first on robust analytical validity. Biomarker assays must demonstrate reproducibility, precision, sensitivity, specificity, and stability across laboratories and patient populations. However, substantial methodological heterogeneity currently exists among studies evaluating inflammatory mediators, circulating molecular markers, and epigenetic signatures [140,141,142].

For epigenetic biomarkers in particular, variability in DNA extraction methods, sample processing, normalization strategies, methylation quantification platforms, and bioinformatic pipelines may significantly influence reported results. Similarly, circulating inflammatory markers may be affected by preanalytical variables such as sample timing, storage conditions, and concurrent systemic inflammatory states [143,144,145].

Assay standardization therefore represents a critical prerequisite for clinical translation. Without harmonized analytical protocols and validated thresholds, comparisons across studies remain difficult, limiting reproducibility and preventing integration into routine diagnostic workflows [140,141,142] (Table 3).

### 8.2. Clinical Validity and Context of Use

Beyond analytical performance, candidate biomarkers must demonstrate clinical validity within clearly defined contexts of use (COU). In prostate disease, clinically relevant contexts may include discrimination between benign inflammatory PSA elevation and clinically significant malignancy, support for biopsy decision-making, progression prediction during active surveillance, or identification of biologically aggressive disease phenotypes [133,134,135,137,138].

Importantly, statistical association alone does not establish clinical validity. Biomarkers must demonstrate reproducible and clinically meaningful relationships with relevant endpoints across independent patient cohorts. Moreover, the biological interpretation of inflammatory and epigenetic biomarkers may vary substantially depending on disease stage, age, comorbidities, medication exposure, and systemic inflammatory burden [141,142,143].

This issue is particularly relevant for biomarkers reflecting systemic immune activity or chronic inflammatory remodeling. Such markers may capture biologically important processes while simultaneously lacking disease specificity, thereby complicating interpretation in multimorbid patient populations frequently encountered in prostate disease management [144,145,146].

### 8.3. Clinical Utility and Incremental Value Beyond Existing Tools

Even analytically robust and clinically associated biomarkers may ultimately fail to demonstrate clinical utility. To justify implementation in prostate diagnostics, candidate biomarkers must provide incremental value beyond existing tools such as PSA, PSA density, PHI, 4Kscore, SelectMDx, risk calculators, and mpMRI [61,62,63,64,65,66,67,68,69,70,71,72,73,74,75,76,77,78,79,80,81,82,134,136].

Clinical utility may involve improved discrimination of clinically significant disease, reduction of unnecessary biopsies, refinement of active surveillance strategies, or improved prediction of progression risk. However, demonstrating such utility requires carefully designed prospective studies evaluating whether biomarker-guided decision-making meaningfully improves patient outcomes or clinical workflows [133,134,135,137,138,147].

For inflammatory and epigenetic biomarkers specifically, an important challenge lies in determining whether systemic biological signals provide sufficiently independent information beyond imaging findings and established molecular assays. Biological plausibility alone is insufficient for implementation without evidence of measurable diagnostic or prognostic improvement [140,141,142,143,144,145,146,147].

### 8.4. Confounding Variables and Biological Complexity

A major challenge in inflammatory and epigenetic biomarker research involves the influence of confounding variables. Age, obesity, smoking, metabolic disease, cardiovascular pathology, medication use, infections, and chronic inflammatory conditions may substantially affect circulating inflammatory mediators and epigenetic profiles independent of prostate disease itself [143,144,145,146].

Similarly, leukocyte-based epigenetic signatures may be influenced by immune-cell composition, immunosenescence, environmental exposure, and systemic inflammatory activity. These factors complicate interpretation and increase the risk of identifying nonspecific systemic signals rather than prostate-related biological alterations [121,122,123,124].

The biological heterogeneity of prostate disease further complicates biomarker development. Benign hyperplasia, chronic inflammation, indolent cancer, and aggressive disease may coexist within the same patient, creating overlapping molecular and inflammatory profiles that challenge binary diagnostic classification models [113,114,115,116].

### 8.5. Need for Prospective Multimodal Validation Cohorts

Future progress in biomarker translation will require large, prospective, well-characterized prostate cohorts integrating clinical, imaging, inflammatory, molecular, and epigenetic data. Such studies should prioritize standardized sample collection, harmonized analytical methodologies, predefined clinical endpoints, and external validation strategies [140,141,142,143,144,145,146,147].

Importantly, biomarker validation should increasingly focus on multimodal biological integration rather than isolated marker performance. Because prostate disease progression emerges from interactions among epithelial transformation, stromal remodeling, immune activation, and systemic biological adaptation, integrative biomarker models may ultimately provide greater clinical relevance than single-marker approaches alone [137,138,140,141,142,143,144,145,146,147].

At present, inflammatory and epigenetic biomarkers in prostate disease remain promising but largely investigational. Their future clinical role will depend on rigorous validation demonstrating analytical robustness, reproducible clinical validity, and measurable incremental utility within clearly defined diagnostic and prognostic contexts.

### 8.6. Structured Narrative Review Approach

This article was designed as a structured narrative review and does not present original experimental data, patient-level data, or a formal systematic review/meta-analysis. Relevant literature was identified through targeted searches of PubMed, Scopus, and Web of Science, with priority given to studies published between 2015 and 2025. Earlier landmark studies were included when they provided essential biological, methodological, or clinical context.

The selection of literature was guided by scientific relevance to inflammatory and epigenetic biomarkers in prostate disease, with particular attention to chronic inflammation, YKL-40/CHI3L1, mannose-binding lectin, MBL2-related innate immune variability, DNA methylation, DNA hydroxymethylation, PSA-based diagnostics, molecular assays, and multiparametric MRI. Higher-confidence evidence was defined as evidence derived from human cohorts, prospective or externally validated studies, systematic reviews, meta-analyses, or studies demonstrating clear mechanistic and translational relevance. When conflicting evidence was identified, greater interpretive weight was assigned to studies with larger cohorts, stronger methodological design, independent validation, and clearer clinical contexts of use.

The included literature was critically interpreted by the authors to provide a translational overview of current evidence, clinical limitations, and future multimarker strategies in prostate disease diagnostics.

### 8.7. Use of Generative Artificial Intelligence Tools

During the preparation of this narrative review, the authors used ChatGPT (OpenAI, GPT-5 series, San Francisco, CA, USA) for language refinement, structural editing support, and preliminary graphical concept development.

The GenAI tool was not used for data collection, data analysis, study design, formal study selection, quality appraisal, interpretation of scientific evidence, or formulation of scientific conclusions. All scientific content, references, interpretations, figures, and final manuscript sections were critically reviewed, verified, modified where necessary, and approved by the authors.

## 9. Conclusions

Chronic inflammation, innate immune modulation, and epigenetic remodeling appear to contribute substantially to prostate disease heterogeneity and progression. Within this context, biomarkers such as YKL-40, mannose-binding lectin, and global DNA methylation/hydroxymethylation represent biologically plausible candidates for improving molecular characterization beyond conventional PSA-based assessment.

However, inflammatory and epigenetic biomarkers should currently be viewed as biologically informative adjunctive layers rather than validated standalone diagnostic tools in prostate disease. Existing evidence remains exploratory, and further prospective validation is required to determine their incremental clinical value beyond PSA, molecular assays, and mpMRI.

Future progress will likely depend on integrative multimarker models combining imaging, molecular, inflammatory, and epigenetic data within clearly defined clinical contexts of use.

## Figures and Tables

**Table 1:** Current diagnostic tools and established/emerging biomarkers in prostate disease.

Tool/Biomarker	Sample/Modality	Main Clinical Role	Key Reported Value	Main Limitation	Evidence Level	Context of Use
PSA	Serum	Initial risk assessment, screening, monitoring	Widely available; cornerstone of prostate evaluation	Low specificity; elevated in BPH, prostatitis, retention, instrumentation	Routine	Initial assessment, screening, monitoring
% free PSA	Serum	Improves discrimination in intermediate PSA range	Lower %fPSA associated with increased PCa probability	Overlap between benign and malignant disease	Validated adjunct	Biopsy decision support
PSA density	Serum + prostate volume	Risk refinement, especially with MRI findings	Improves interpretation of PSA in enlarged prostates	Depends on accurate prostate volume measurement	Validated adjunct	Biopsy decision support, risk stratification
PHI	Serum	Detection of clinically significant PCa	Better specificity than PSA alone; useful adjunct before biopsy	Cut-offs vary; performance depends on population and MRI context	Validated adjunct	Biopsy decision support, csPCa risk assessment
4Kscore	Serum	Estimation of high-grade PCa risk	Combines kallikrein markers and clinical variables	Not a standalone test; requires contextual interpretation	Validated adjunct	Biopsy decision support, high-grade PCa risk estimation
PCA3	Post-DRE urine	Adjunctive PCa detection, especially repeat-biopsy setting	More cancer-specific than PSA	Limited ability to define aggressiveness alone	Validated adjunct	Repeat-biopsy decision support
SelectMDx	Urine mRNA assay	Identification of men at risk for clinically significant PCa	Prospective data support use with or without mpMRI in biopsy-naïve men	Performance depends on pathway and population	Validated adjunct	Biopsy decision support, csPCa risk stratification
TruBlood/CTC-based tests	Blood	Emerging liquid biopsy approach	Preliminary studies suggest diagnostic potential	Investigational; requires large external validation	Investigational	Liquid biopsy, risk stratification
mpMRI/PI-RADS	Imaging	Lesion detection, targeted biopsy, biopsy avoidance	Improves detection of clinically significant PCa and reduces unnecessary biopsy	False negatives occur; reader expertise and PI-RADS 3 uncertainty remain	Routine	Lesion detection, biopsy targeting, risk stratification
Risk calculators	Clinical + biomarkers ± MRI	Individualized biopsy decision support	Integrate multiple variables for risk-adapted decisions	Calibration varies across populations	Validated adjunct	Personalized biopsy decision support

Conventional PSA-based assessment has progressively evolved toward integrated diagnostic pathways combining PSA derivatives, blood- and urine-based molecular assays, mpMRI, and risk calculators. These tools improve risk stratification but incompletely capture inflammatory remodeling, immune variability, and long-term molecular adaptation.

**Table 2:** Candidate inflammatory and epigenetic biomarker layers in prostate disease.

Biomarker	Biological Layer Reflected	Potential Context of Use	Current Evidence Status	Main Limitation	Context of Use	Evidence Level
YKL-40/CHI3L1	Macrophage activation, ECM remodeling, angiogenesis, inflammatory stromal activity	Characterization of inflammatory or remodeling-associated phenotypes; possible adjunct in risk models	Biologically plausible; prostate-specific evidence limited and exploratory	Not prostate-specific; affected by systemic inflammation, cancer, cardiovascular and pulmonary disease	Inflammation vs. csPCa discrimination; inflammatory phenotype characterization; risk stratification	Investigational
MBL	Innate immune recognition, lectin complement pathway activation	Assessment of innate immune variability and inflammatory susceptibility	Mechanistic rationale; direct prostate validation scarce	Strong genetic and population variability; context-dependent effects	Innate immune profiling; inflammatory susceptibility assessment	Investigational
MBL2 polymorphisms	Genetically determined innate immune variability	Exploratory inflammatory-risk profiling	Supported in immune/inflammatory diseases; prostate translation hypothetical	Not validated for prostate diagnosis or prognosis	Inflammatory risk profiling; biological phenotyping	Investigational
Global DNAm	Long-term epigenetic imprint, gene regulation, inflammatory exposure	Risk stratification, molecular complement to PSA/MRI, possible surveillance support	Strong biological rationale; prostate and liquid biopsy evidence emerging	Influenced by age, leukocyte composition, lifestyle, comorbidities	Risk stratification; molecular complement to PSA/mpMRI; active surveillance support	Advanced investigational
Global DNAhm	Dynamic epigenetic regulation, TET-related demethylation activity	Early molecular dysregulation; systemic immune-state readout	Exploratory prostate evidence; promising but limited	Assay complexity; limited standardized thresholds	Early molecular dysregulation assessment; biological phenotyping	Investigational
ctDNA methylation markers	Tumor-associated liquid biopsy signal	Detection, prognosis, monitoring	Growing evidence in prostate and other cancers	Sensitivity may vary by tumor burden and disease stage	Detection; prognosis; monitoring; active surveillance support	Advanced investigational

These markers are not presented as validated standalone diagnostic tools, but as biologically informative adjunctive layers that may complement PSA, molecular assays, and mpMRI in future multimarker models.

**Table 3:** Validation requirements and translational challenges for inflammatory and epigenetic biomarkers.

Validation Domain	Requirement	Specific Challenge in Prostate Disease	Practical Implication
Analytical validity	Reproducible and precise measurement	Different platforms, sample handling, normalization methods	Harmonized protocols and inter-laboratory validation are required
Clinical validity	Association with relevant endpoints	BPH, prostatitis, indolent PCa, and aggressive PCa may overlap biologically	Endpoints must be clearly defined: csPCa, biopsy result, progression, treatment response
Clinical utility	Improvement in decision-making	Biomarker must add value beyond PSA, PSAD, PHI, 4Kscore, SelectMDx, mpMRI	Incremental value should be tested using prediction models and decision curve analysis
Confounding control	Adjustment for non-prostate influences	Age, obesity, infections, smoking, cardiovascular disease, medication use	Cohorts need detailed clinical metadata
Biological specificity	Distinguish prostate-related signal from systemic inflammation	YKL-40, MBL, DNAm, and DNAhm are not organ-specific	Best used in panels, not alone
Prospective validation	Testing in independent cohorts	Many studies remain exploratory or retrospective	Large prostate-specific prospective cohorts are needed
Context of use	Define exact clinical question	Elevated PSA? Biopsy decision? Active surveillance? Progression risk?	Each biomarker may require a different validation pathway

Candidate biomarkers must demonstrate analytical validity, clinical validity, and clinical utility within clearly defined contexts of use before clinical implementation can be considered. Mere biological plausibility or statistical association is insufficient for diagnostic adoption.

## Data Availability

No new data were created or analyzed in this study.

## Abbreviations

BPH	Benign prostatic hyperplasia
CHI3L1	Chitinase-3-like protein 1
COU	Context of use
COPD	Chronic obstructive pulmonary disease
CRP	C-reactive protein
csPCa	Clinically significant prostate cancer
ctDNA	Circulating tumor DNA
DNA	Deoxyribonucleic acid
DNAm	DNA methylation
DNAhm	DNA hydroxymethylation
DRE	Digital rectal examination
ECM	Extracellular matrix
EWAS	Epigenome-wide association study
IgE	Immunoglobulin E
IL-6	Interleukin-6
JAK/STAT	Janus kinase/signal transducer and activator of transcription
MAPK	Mitogen-activated protein kinase
MBL	Mannose-binding lectin
MBL2	Mannose-binding lectin 2 gene
mpMRI	Multiparametric magnetic resonance imaging
MRI	Magnetic resonance imaging
NF-κB	Nuclear factor kappa B
PCA3	Prostate cancer antigen 3
PCa	Prostate cancer
PHI	Prostate Health Index
PI-RADS	Prostate Imaging Reporting and Data System
PSA	Prostate-specific antigen
PSAD	Prostate-specific antigen density
TET	Ten-eleven translocation
TNF-α	Tumor necrosis factor alpha
WBC	White blood cell
YKL-40	Chitinase-3-like protein 1 (cartilage glycoprotein-39)
